# Evaluation of the accessibility and its equity of the national public-private mix program for tuberculosis in Korea: a multilevel analysis

**DOI:** 10.4178/epih.e2023002

**Published:** 2022-12-07

**Authors:** Hyunjin Son, Changhoon Kim

**Affiliations:** 1 Department of Preventive Medicine, Dong-A University College of Medicine, Busan, Korea; 2 Busan Center for infectious Disease Control and Prevention, Pusan National University Hospital, Busan, Korea; 3 Department of Preventive Medicine, Pusan National University College of Medicine, Busan, Korea

**Keywords:** Tuberculosis, Health disparities, Social determinants, Accessibility, Equity

## Abstract

**OBJECTIVES:**

To examine the effect of individual and area-level characteristics on the probability of public-private mix (PPM) support (PPM coverage) for tuberculosis (TB).

**METHODS:**

This study is a retrospective cohort design using TB reporting and treatment management data in Korea. We analyzed PPM coverage through multilevel logistic regression and empirical Bayesian estimation according to individual and area-level characteristics and their interaction.

**RESULTS:**

Patients aged 0-29 years, women, of Korean nationality, treated at a general hospital, a one-time reporting, urban areas, and the lowest deprivation index (DI) showed higher PPM coverage. Due to the cross-level interaction, PPM coverage in the urban areas (slope=-0.048, p<0.001) had a higher level but a steeper negative deprivation gradient than in rural areas (slope= -0.015, p<0.001). For a general hospital, the PPM coverage in urban is high but more significantly decreased than in rural areas with the higher DI (urban: slope=-0.047, p<0.001; rural: slope=-0.031, p<0.001). For clinics and hospitals, the effect of DI did not appear in urban areas, but in rural areas, the higher the DI, the higher the PPM coverage with a slope of 0.046 (p<0.001) and 0.063 (p<0.001), respectively.

**CONCLUSIONS:**

The PPM program created a significant disparity in PPM coverage between urban-rural areas and type of healthcare provider according to DI. Considering the high risk of TB incidence in areas with higher DI, institutional improvement and program redesign are needed to improve accessibility and equity.

## GRAPHICAL ABSTRACT

[Fig f3-epih-45-e2023002]

## INTRODUCTION

The Korean government has considered tuberculosis (TB) a significant public health problem. It developed a National Tuberculosis Program (NTP) in 2006 and a five-year National TB strategic plan in 2013. The World Health Organization (WHO) has been utilizing the public-private mix (PPM) strategy as a core tool to promote access to quality TB care, including Directly Observed-Treatment short-course (DOTS). WHO has expanded its overall TB management capacity by increasing the participation of the private sector worldwide since 2001. Being affected by this strategy, the Korean NTP initiated a hospital-based PPM program (Supplementary Material 1) that, however, differs from WHO’s strategy of “Engaging all healthcare providers (HCPs) in TB control “, which considers promoting access to quality TB care as essential.

The PPM approach, which aims to engage all HCPs, has four objectives (to increase the finding of TB cases, to improve TB treatment outcomes, to improve accessibility and equity, and to reduce the financial burden of patients) [5]. There were studies on improving TB treatment outcomes in relation to the PPM program in Korea [4,6], not yet research on accessibility and equity. TB has a significant social disparity between countries, between areas and has the highest risk to the poorest [7]. If regions with a high incidence risk have poor accessibility and quality of treatment, the disparity is bound to widen. Thus, to systematically evaluate the program and suggest improvement strategies, it is necessary to study whether there is a disparity in accessibility and quality and the associated factors in a model that considers individual and area characteristics simultaneously.

An area is a significant factor in the prevention and management of TB. The studies conducted in high-income and low-income countries setting have identified the association between area-level deprivation and social risk factors (poor housing conditions, high unemployment, regional migration rates, low household income, high inequality, and a composite index of poverty, etc.) and TB using traditional ecological analysis and geographic information system (GIS) [8]. Multilevel analysis is of great importance in infectious disease research because it can determine whether individuals and area-level characteristics have an effect and interactions [9]. The researchers conducted multilevel studies on TB incidence and reporting [8,10], transmission [11], adherence to guidelines for diagnosis and treatment [12], spatial distribution patterns [13], and distribution of multiple drug resistance (MDR) TB [14]. The material and collective social function of the area determines the distribution of HCPs and the accessibility to TB HCPs, and public health functions such as the TB control program of the public health center (PHC) influence treatment support as a collective social function of the area [15]. Since the area can affect health through material infrastructure and collective social function apart from compositional effects of residents, if properly operationalized, we can test the causal hypothesis linking the area and health outcome.

This study aimed to examine the probability of PPM support (PPM coverage) from the accessibility and equity perspective, one of the objectives of the PPM program, by conducting a multilevel analysis.

## MATERIALS AND METHODS

### Subjects

This study is a retrospective cohort design using TB reporting and treatment management data in Korea. We received data on new TB patents from 2012 to 2016 from the Korea Disease Control and Prevention Agency in July 2017. TB reporting data included the address of patients identified by PHCs were living, which we used to link with area-level variables, and treatment management data recorded the types of HCPs that provided treatment. Because the treatment of new TB patients lasts more than six months, we included new TB patients notified from 2012 to 2015 for the study subjects. We included 19,308 patients with more than one reporting from different HCPs as single patients. We excluded 1,810 patients with rifampicin resistance (RR) or MDR. Finally, the study subjects were a cohort of 137,865 drugsusceptible new TB patients, excluding 36 patients with missing values in the analysis variables.

### Definition of variables and data processing

We defined the individual-level outcome variable, whether the PPM program support TB treatment, that the HCP where the patient selected after receiving PPM program information on reporting was a PHC or a PPM HCP supervised by a TB specialty nurse.

Individual-level explanatory variables included year (2012, 2013, 2014, and 2015), age group (0-29, 30-64, and ≥ 65), gender, nationality (Korean and immigrant), type of HCP (clinics including PHCs, general hospitals, and hospitals), type of TB (extrapulmonary TB, smear-positive pulmonary TB, and smear-negative pulmonary TB), and the number of reporting (1, 2, and ≥ 3). Although clinics and PHCs have different characteristics, they consolidated in analysis considering that they function as primary HCP with high accessibility to TB patients. We used districts (si, gun, and gu) according to the individual residence as the area unit, which is administratively defined and the smallest statistical unit for which official data are available (there are 252 PHCs, but only 245 areas were included in this study considering the changes in administrative districts) and have the authority of independent policy implementation. The area-level explanatory variables were the deprivation index (DI) and urbanicity. By applying the method [16,17] used in other studies, we calculated DI using the 2010 Population and Housing Census samples. A higher DI value indicates a higher deprivation status. The urban areas included gun and gu in the metropolitan area and si in the provincial area, and the rural areas included the other areas.

### Statistical analysis

Multilevel logistic regression analysis for PPM coverage estimated a series of models from simple to complex. We examined area-level variation with the “empty” model with only random intercept. Then the model included the individual-level variables to investigate how the difference in the individual composition explains area-level variation. To investigate whether the area-level variables conditioned area-level variation, we examined the degree of explanation of area-level variation, including the DI, urbanicity, and the interaction between these variables and the type of HCP [18,19].

We presented the values of the median odds ratio (MOR) and the interval odds ratio (IOR) of 80% (IOR-80) to directly compare area-level variation with the odds ratio (OR) of the individual variables. MOR was the value of quantifying heterogeneity among groups (cluster effect). IOR-80 was the value of quantifying the area-level covariate effect by considering the area-level fixed effect and the random residual variation [20,21]. To visualize the complex relationship between DI, urbanicity, and type of HCP at the area level, we presented the area-specific estimates by empirical Bayesian (EB) estimation in a graph. The EB estimation has the advantage of improving estimates of parameters for a given group by combining information from the given group with information from all other groups [18,22].

When calculating area-specific estimates for model 2, we designated the fixed effect for individual-level variables as 2015, 30-64 years old, women, smear-positive pulmonary TB, Korean nationality, and one reported case, general hospital, respectively. We included random effect of urbanicity and DI. For model 3, we designated the variables for the fixed effect as the same except for the type of HCP. We included the random effect of the DI and urbanicity by the type of HCP (We presented the results for other ages, gender, and nationality in the Supplementary Material 2).

**Figure 1.**
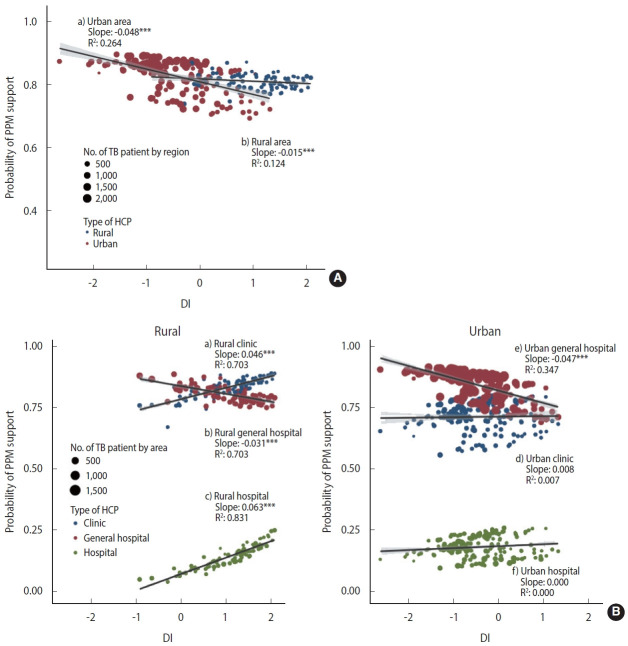
Area-specific probability of public-private mix (PPM) support according to urbanicity, deprivation index (DI), and type of healthcare provider (HCP). The empirical Bayesian method estimates the area-specific probability of PPM support with (A) model 2, which includes the 2-way interaction effect of urbanicity and DI, and (B) model 3, which includes the 3-way cross-level interaction effect of urbanicity, DI, and type of HCP. To consider the effect of the patient size by area, we performed a weighted regression analysis in which the number of patients weighted the area-specific estimates by the area in the corresponding group. The regression coefficient represents the relationship between deprivation, urbanicity, and PPM support (1-(A)) and the relationship between deprivation, urbanicity, type of HCP, and PPM support (1-(B)). TB, tuberculosis.

There is evidence that the additive model can reflect more reality than the multiplicative model in evaluating epidemiological interactions [23]. To reflect the effect of patient size by area, we performed a weighted regression analysis using weights with the numbers of patients by area. Stratified by urbanicity, we presented the relationship between the DI and the PPM coverage (Figure 1A) and the relationship between the DI and PPM coverage considering the interaction with the type of HCP (Figure 1B) with regression coefficients.

**Figure 2.**
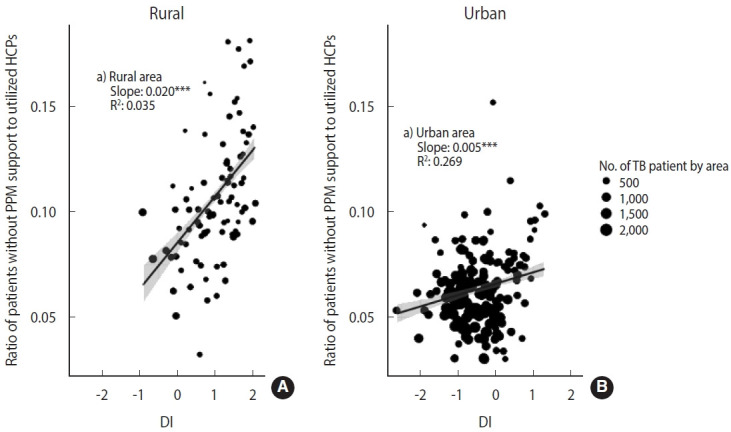
The ratio of patients without public-private mix (PPM) support to utilized healthcare providers (HCPs) according to urbanicity (A: rural, B: urban) and deprivation index (DI). To consider the effect of the patient size by area, we perform a weighted regression analysis in which the number of patients weighted the area-specific estimate in the corresponding group. The regression coefficient represents the relationship between the DI, urbanicity, and ratio of non-PPM support to HCPs. We estimated the ratio of patients who did not have PPM support to the number of HCPs utilized by area according to urbanicity and deprivation. TB, tuberculosis.

We also presented area-level characteristics of HCPs that patients without PPM support utilized (due to reasons such as inability to use participating HCPs or reluctance to receive treatment at PHCs, etc.) (Figure 2). We estimated the ratio of patients without PPM support to the number of utilized HCPs according to urbanicity and DI. Like in Figure 1, we conducted a weighted regression analysis using weights with the number of patients by area.

### Ethics statement

The protocol was deliberated by the Institutional Review Board of Pusan National University Hospital (IRB approval No. H-1708-030-058). The obligation for the explanation and informed consent was waived in recognition of the respective cohort study conducted with data regularly collected. Data were anonymized in advance and then analyzed.

## RESULTS

### Characteristics of the subjects

Of the 137,865 people included in the study, the earliest year (27.7% in 2012), 30-64 years for age group (48.9%), men (57.3%), and Korean nationality (96.1%) accounted for the highest proportions. 79.1% had pulmonary TB, and 20.9% had extrapulmonary TB. General hospitals by the type of HCP (77.1%), one-time reporting by the number of reporting (86.0%), and urban areas by urbanicity (87.2%) accounted for the highest proportions.

### Public-private mix coverage

The overall PPM coverage was 76.0% (95% confidence interval [CI], 75.8 to 76.2). No statistically significant differences and trends were seen by year in 2012-2016. For individual-level variables, patients aged 0-29 years (79.2%), women (77.3%), Korean nationality (76.1%), treated at general hospitals (84.0%), and one-time reporting (76.7%) had higher PPM coverage at statistically significant levels. As for the area-level variables, the urban area (77.1%) and the lowest quintile DI (80.7%) showed higher PPM coverage (Table 1).

Table 2 presents the fixed and random parameters estimated in the multilevel logistic regression analysis. From the empty model (Model 0) to the model that included individual-level variables, area-level variables, and cross-level interactions (Model 3), the CI of the MOR values for the PPM coverage did not have 1, indicating a significant area-specific difference, but area-level heterogeneity (cluster effect) was not considerable at 1.004. Even when we included additional individual-level and area-level variables, there was no significant change in area-level heterogeneity. In the case of adding individual-level variables (Model 1), there was a statistically significant relationship in all variables except for the year, as in Table 1. The area-level variation decreased by 20%, which means that the difference in the composition of individual-level variables explains 20% of the area-level variation. Model 2 with DI and urbanicity as area-level variables showed area-level variation was slightly reduced (-1.4%), but a similar level of cluster effect as in model 1 (not shown in the table, but 4.8% increase in variation when including urbanicity and -5.9% decrease in variation when including DI). As for the fixed effect of area-level variables, the OR of PPM coverage decreased in an urban and higher DI areas. The IOR-80 estimates of urbanicity and DI did not include 1 in the IOR-80 interval value, and the range was narrow, which means that although the cluster effect of the PPM support was not large, urbanicity and DI explain a significant part of the variation.

We identified the two-way interaction between urbanicity and DI in model 2 and the three-way cross-level interaction between urbanicity, DI, and type of HCP in model 3. Table 3 summarizes the results. The interaction effect estimated from model 2 showed that the OR of PPM support was lower in urban areas than in rural areas (OR, 0.93). The higher the DI, the higher the OR of PPM support in rural areas (OR, 1.15) and the lower OR of PPM support in urban areas (OR, 0.84). The interaction effect estimated from model 3 showed that urban areas were more likely to receive PPM support than rural areas even if they used the same hospital (OR: hospital urban/hospital rural= 2.67) and general hospitals and clinics (including PHCs) were more significantly likely to receive PPM support than hospitals. However, the level was lower in urban areas (OR: clinic urban/hospital rural= 30.76; general hospital urban/hospital rural = 57.61) than in rural areas (OR: clinic rural/hospital rural= 46.09; general hospital rural/hospital rural= 66.37). The slopes according to DI were also different according to the type of HCP and urbanicity. In the case of general hospitals, the higher the DI, the lower the OR of the PPM support, and the level were much lower in urban areas (OR, 0.77) than in rural areas (OR, 0.98). In the case of clinics and hospitals, the higher the DI, the higher OR of the PPM support, and the values were the order of rural hospitals (OR, 2.30), rural clinics (OR, 1.68), city hospitals (OR. 1.16), and city clinics (OR, 1.12).

Figure 1A shows the effect of urbanicity and DI on the PPM coverage, for which we estimated area-specific estimates by applying the empirical Bayesian (EB) estimation method to model 2. In the distribution of DI, the rural areas had higher values, and the area-specific PPM coverage tended to decrease with the higher DI. In rural areas, multilevel logistic regression analysis showed that the higher the DI, the higher the OR of PPM support. However, the EB estimation and weighted regression at the area-level showed that the larger DI, the lower the PPM coverage. The slope of the decline was steeper in urban areas (slope= -0.048, p< 0.001), which showed negative associations on both scales, compared to rural areas (slope=-0.015, p< 0.001).

Figure 1B visualizes the three-way cross-level interaction effect of the deprivation, type of HCP, and urbanicity on the PPM coverage calculated with EB estimation by applying model 3. When receiving treatment at a general hospital, the PPM coverage in urban is higher but more significantly decreased than in rural areas with the higher DI (urban: slope= -0.047, p< 0.001; rural: slope= -0.031, p< 0.001). When receiving treatment at clinics and hospitals, the effect of DI did not appear in urban areas, but in rural areas, the higher the DI, the higher the PPM coverage with a slope of 0.046 (p< 0.001) and 0.063 (p< 0.001), respectively, which have compensated for the decrease in the PPM coverage at a general hospital with the higher DI.

Figure 2 shows the ratio of patients who did not have PPM support by area to the number of HCPs utilized according to urbanicity and DI. Although many patients did not receive PPM support in urban areas, the value of the ratio (median= 0.09 in rural areas; median=0.06 in urban areas) and the slope according to DI (slope= 0.020 in rural areas, p< 0.001; slope= 0.005, p< 0.001 in urban areas) was prominent in rural areas.

This result indicates that the PPM program based on hospitals with a large caseload created a situation where there need to be more available PPM HCPs in rural areas. Therefore, the residents of rural areas have no choice but to select non-PPM HCPs when choosing PPM HCPs in urban areas is challenging. The pattern is proportionally related to DI. The PPM program is widening the gap according to urbanicity and DI in TB quality care.

## DISCUSSION

This study utilized multilevel analysis to evaluate the performance and limitations of the operation of the hospital-based PPM program in terms of accessibility and equity, which consisted of 120 hospitals and 252 PHCs in the early stage of the program (in 2012-2015). These results can help identify the causal pathways and mechanisms of individual and area-level factors for treatment outcomes (success, failure, death, etc.), which are the primary indicators of national TB management, and help develop a regional strategy for TB eradication.

Since 2011, the nationwide TB PPM Program has increased the overall level of PPM coverage (76% overall). However, it has created a PPM coverage disparity with a significant slope according to DI as a result of the three-way interaction between urbanicity, deprivation, and choosing of HCPs (slope for urban areas, -0.048, p< 0.001; slope for rural areas, -0.015, p< 0.001). Given the high TB risk in areas with high DI, there is a need for institutional improvement and program redesign to improve accessibility and equity.

### Implications for public health

Even if all PHCs participate in the PPM program and provide referrals and direct support, it takes work to provide sufficient and equitable PPM support. In the TB PPM program in Korea, the number of participating HCPs is small, and by 2015, only 31.7% (80 out of 252) of area units have participating HCPs (primarily urban areas, Supplementary Material 1). Applying the results of this study, each group has a PPM coverage distribution of 62.9-86.6% for the women 30-64-year-old age group, 55.7-82.1% for the men 65-year-old age group, and 52.6-80.9% for migrants (Supplementary Material 2). Considering the disparity between area-levels deprivation and the individual-level variables such as the elderly and migrants, some groups only appear to receive PPM support at about 50%, indicating a severe problem of equity in accessibility.

Although the treatment success rate in the private sector has improved from 70.3% to 83.9% over five years since the introduction of the nationwide PPM program [4], this is likely to be the effect of more patients referred to the large private HCPs involved in the program, which have a high treatment success rate. There needs to be more evidence to expect overall performance improvement in a situation where central and local health authorities (including PPM programs) do not have a system to regulate and induce private HCPs to follow TB treatment guidelines or link them to public health functions. It is necessary to shift from the “strategy for referral to program participating HCPs” to the “strategy for engaging all HCPs” at the PPM program, as recommended by the WHO. If the current PPM program continues, the treatment success rate (when applying, treatment success rate ORs [6]: PPM general hospital 1, non-PPM general hospital 0.66, PPM hospital 0.64, non-PPM hospital 0.49, and clinic 0.39) would be low for areas and population groups that do not receive sufficient PPM support.

When health authority provided the DOTS from a place near patients through the intensive decentralization of care, the cost of travel and opportunity became the minimum, increasing the patient’s adherence [25]. In addition, when the PPM program engaged HCPs or had programs to provide services in poor areas, the equity of access increased [26]. These are crucial evidence for “engaging all HCPs.” In addition to these policies, Korean NTP should consider introducing an approach for utilizing local data [27] or control efforts tailored to local conditions [10-13], such as cohort review that allows for systematic monitoring of courses and outcome indicators within the area. Without NTP’s additional control efforts for the social determinant of TB, the PPM program could lead to the unintended widening of health inequalities [28,29].

### Methodological issues

This study has several strengths from a methodological point of view. First, we evaluated PPM coverage using individual PPM support as the outcome variable instead of simply utilizing the number or availability of TB HCPs by population group for each area as an indicator and in-depth accessibility and equity from the perspective of universal health coverage [1-3]. Second, we analyzed patients registered for TB for the stabilized period after the complete introduction of the PPM program, by which we ensured a large sample size and representativeness. Third, considering the variables at the individual and the area-level simultaneously using the multilevel models, we examined the extent to which each of the variables and their interaction explains the area-level variation on the PPM coverage. It is a conditioned choice that the effect of area unit, urbanicity, and deprivation affects the type of HCP for treatment. Therefore, we did not use GIS directly but could show the effect of accessibility to the PPM support. Area-specific accessibility indicators would be calculated in subsequent studies using the information on individual residences and the location of hospitals and clinics.

Several limitations also exist. First, the TB reporting in Korea does not collect variables for individual socioeconomic positions not included in the WHO guidelines [5], so we could not analyze it in this study. The result cannot exclude the possibility of residual confounding because we did not consider the effect of the socioeconomic status positions of the individual in the models. However, residual confounding is likely to affect only the magnitude of the effect so that it will have a minimal impact on the overall interpretation of the results of this study. Second, although the degree of variation according to the area unit (gu and gun) was statistically significant, it was not large. However, Other studies don’t recommend determining the adequacy of the model to the extent of variation explained by the area unit using administrative regions [19,30]. In a situation where only a few HCPs participate in the PPM program, the activities of health authorities affect the choice of treatment, such as referring to HCPs or providing direct care. Therefore, we can consider area units to reflect the collective social attributes formed by the policies. In follow-up studies, it is necessary to investigate the relative importance and magnitude of the effects by implementing analytical studies on the cross-classified structure that additionally include the service area of PPM HCPs [31].

## Figures and Tables

**Figure f3-epih-45-e2023002:**
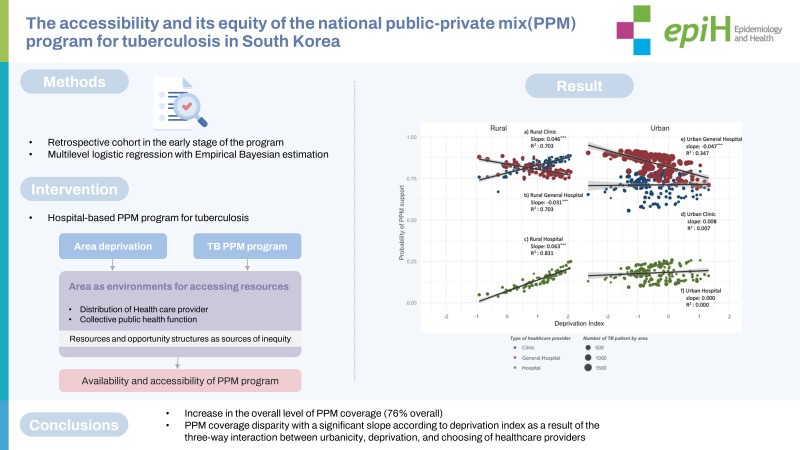


**Table 1.** The probability of PPM support (PPM coverage) according to study characteristics

Characteristics			Study population, n (%)	p-value^1^	PPM coverage, % (95% CI)	p-value^1^
Total			137,865 (100)		76.0 (75.8, 76.2)	
Individual-level variable				<0.001		0.153
	Year					
		2012	38,138 (27.7)		75.6 (75.2, 76.1)	
		2013	35,156 (25.5)		76.1 (75.7, 76.6)	
		2014	33,877 (24.6)		76.3 (75.9, 76.8)	
		2015	30,694 (22.3)		75.9 (75.4, 76.4)	
	Age (yr)			<0.001		<0.001
		0-29	22,836 (16.6)		79.2 (78.7, 79.7)	
		30-64	67,466 (48.9)		77.3 (77.0, 77.6)	
		≥65	47,563 (34.5)		72.6 (72.2, 73.0)	
	Gender			<0.001		<0.001
		Men	79,016 (57.3)		75.0 (74.7, 75.3)	
		Women	58,849 (42.7)		77.3 (77.0, 77.6)	
	Nationality			<0.001		<0.001
		Korean	132,431 (96.1)		76.1 (75.8, 76.3)	
		Migrant	5,434 (3.9)		73.8 (72.6, 75.0)	
	Type of healthcare provider			<0.001		<0.001
		Clinic	17,817 (12.9)		73.9 (73.2, 74.5)	
		General hospital	106,350 (77.1)		84.0 (83.7, 84.2)	
		Hospital	13,698 (9.9)		16.8 (16.2, 17.5)	
	Type of TB			<0.001		<0.001
		Extra-pulmonary TB	28,829 (20.9)		79.8 (79.3, 80.3)	
		Pulmonary, smear-TB	41,068 (29.8)		74.4 (73.9, 74.8)	
		Pulmonary, smear+TB	67,968 (49.3)		75.3 (75.0, 75.7)	
	No. of times notified			<0.001		<0.001
		1	118,497 (86.0)		76.7 (76.4, 76.9)	
		2	16,275 (11.8)		72.8 (72.1, 73.5)	
		≥3	3,093 (2.2)		66.1 (64.4, 67.8)	
Area-level variable						
	Urbanicity			<0.001		<0.001
		Rural	17,622 (12.8)		68.7 (68.0, 69.4)	
		Urban	120,243 (87.2)		77.1 (76.8, 77.3)	
	Deprivation index			<0.001		<0.001
		1st (least)	27,584 (20.0)		80.7 (80.2, 81.1)	
		2nd	27,878 (20.2)		79.2 (78.8, 79.7)	
		3rd	28,150 (20.4)		75.3 (74.8, 75.8)	
		4th	26,732 (19.4)		76.4 (75.9, 76.9)	
		5th (most)	27,521 (20.0)		68.3 (67.7, 68.8)	

PPM, public-private mix; CI, confidence interval; TB, tuberculosis.

1 Using by χ^2^ test.

**Table 2.** Parameter estimates from multilevel logistic regression model

Variables				Model 0	Model 1	Model 2	Model 3
Fixed effect							
	Individual level						
		Year					
			2012		1.00 (reference)	1.00 (reference)	1.00 (reference)
			2013		1.04 (1.00, 1.08)	1.03 (1.00, 1.08)	1.03 (0.99, 1.07)
			2014		1.03 (0.99, 1.07)	1.03 (0.99, 1.07)	1.03 (0.99, 1.07)
			2015		1.03 (0.99, 1.08)	1.03 (0.99, 1.08)	1.03 (0.99, 1.07)
		Age (yr)					
			0-29		1.00 (reference)	1.00 (reference)	1.00 (reference)
			30-64		0.90 (0.87, 0.94)^***^	0.91 (0.87, 0.94)^***^	0.90 (0.86, 0.93)^***^
			≥65		0.75 (0.71, 0.78)^***^	0.75 (0.72, 0.78)^***^	0.74 (0.71, 0.77)^***^
		Gender					
			Men		1.00 (reference)	1.00 (reference)	1.00 (reference)
			Women		1.11 (1.08, 1.14)^***^	1.11 (1.08, 1.14)^***^	1.10 (1.07, 1.14)^***^
		HCP					
			Hospital		1.00 (reference)	1.00 (reference)	1.00 (reference)
			Clinic		13.20 (12.47, 13.9)^***^	13.3 (1.95, 2.11)^***^	46.4 (35.4, 60.9)^***^
			General hospital		26.82 (25.53, 28.1)^***^	27.0 (25.7, 28.3)^***^	66.4 (52.4, 84.0)^***^
		Type of TB					
			Pulmonary, smear+TB		1.00 (reference)	1.00 (reference)	1.00 (reference)
			Extra-pulmonary TB		0.97 (0.93, 1.01)^***^	0.97 (0.93, 1.02)^***^	1.10 (1.06, 1.15)^***^
			Pulmonary, smear-TB		0.90 (0.87, 0.94)^***^	0.90 (0.87, 0.94)^***^	1.08 (1.05, 1.12)^***^
		Nationality					
			Korean		1.00 (reference)	1.00 (reference)	1.00 (reference)
			Migrant		0.81 (0.75, 0.87)^***^	0.81 (0.75, 0.87)^***^	0.82 (0.77, 0.88)^***^
		No. of times notified					
			1		1.00 (reference)	1.00 (reference)	1.00 (reference)
			2		1.47 (1.41, 1.55)^***^	1.48 (1.41, 1.55)^***^	1.43 (1.37, 1.50)^***^
			≥3		1.24 (1.13, 1.36)^***^	1.24 (1.12, 1.36)^***^	1.22 (1.10, 1.34)^***^
	Area level						
		Urbanicity					
			Rural			1.00 (reference)	1.00 (reference)
			Urban			1.09 (1.04, 1.15)^***^	2.67 (2.11, 3.37)^***^
			Deprivation			1.13 (1.08, 1.18)^***^	2.30 (1.96, 2.72)^***^
			Urbanicity × Deprivation				
			Urban × Deprivation			0.74 (0.71,0.78)^***^	0.50 (0.42, 0.61)^***^
	Cross-level						
		HCP × Deprivation					
		Clinic × Deprivation				-	0.73 (0.59, 0.90)^***^
		General hospital × Deprivation				-	0.43 (0.36, 0.51)^***^
		HCP × Urbanicity					
		Clinic × Urban				-	0.25 (0.19, 0.33)^***^
		General hospital × Urban				-	0.33 (0.25, 0.42)^***^
		HCP × Urbanicity × Deprivation					
		Clinic × Urban × Deprivation				-	1.33 (1.04, 1.68)^***^
		General hospital × Urban × Deprivation				-	1.56 (1.28, 1.89)^***^
Random effect							
	σ (95% CI)			0.005 (0.001, 0.017)	0.004 (0.001, 0.017)	0.004 (0.001, 0.017)	0.004 (0.001, 0.017)
	MOR			1.004 (1.001, 1.018)	1.004 (1.001, 1.016)	1.004 (1.001, 1.016)	1.004 (1.001, 1.016)

Values are presented as adjusted odds ratio (95% CI). MOR, median odds ratio; HCP, healthcare provider; TB, tuberculosis; CI, confidence interval.

*** p< 0.001.

**Table 3.** Effect of two-way and three-way interaction between HCP, DI, and urbanicity

Model			Urbanicity		Urbanicity × DI	
			aOR	IOR-80	aOR	IOR-80
Model 2	Rural		Reference	-	1.15 (0.03)^***^	1.15-1.17
	Urban		0.93 (0.03)^***^	0.84-0.85	0.84 (0.03)^***^	0.84-0.85
			HCP × Urbanicity		HCP × Urbanicity × DI	
Model 3	Rural					
		Hospital	Reference		2.30 (0.19)^***^	2.29-2.32
		Clinic	46.09 (6.41)^***^	46.09-46.76	1.68 (0.12)^***^	1.67-1.69
		General hospital	66.37 (7.98)^***^	65.9-66.86	0.98 (0.03)^***^	0.98-0.99
	Urban					
		Hospital	2.67 (0.32)^***^	2.65-2.69	1.16 (0.05)^***^	1.15-1.17
		Clinic	30.76 (3.60)^***^	30.54-30.99	1.12 (0.03)^***^	1.11-1.13
		General hospital	57.61 (6.63)^***^	57.19-58.03	0.77 (0.01)^***^	0.77-0.78

Estimated marginal means for urbanicity and HCP in model 2 and model 3 and comparisons or contrasts among them were estimated; To express the interaction between a continuous variable and two categorical variables, Table 3 presents at the designated factor levels and DI value of 0 the interaction effect of categorical variables (left) and the slope of DI (right). HCP, healthcare provider; DI, deprivation index; aOR, adjusted odds ratio; IOR-80, interval odds ratio of 80%.

*** p<0.001.

## SUPPLEMENTARY MATERIALS

Korean version is available at https://www.e-epih.org/.

Supplementary Material 1. TB-PPM program in South Korea

Supplementary Material 2. Empirical Bayesian estimates using other fixed level values
