# Supplementary material for: Evaluation of the accessibility and its equity of the national public-private mix program for tuberculosis in Korea: a multilevel analysis
Source: Epidemiol Health. 2022 Dec 7;45:e2023002. doi: 10.4178/epih.e2023002 (PMC10266928; doi:10.4178/epih.e2023002)
Supplement: Supplementary file 1 [file epih-45-e2023002-Korean-Supplementary.docx]

**Title**

Evaluation of the accessibility and its equity of the national public-private mix program for tuberculosis in South Korea: a multilevel analysis

**Abstract**

**Objectives: To** examine the effect of individual-level and area-level characteristics on the probability of Public-Private Mix (PPM) program support for tuberculosis (TB) (PPM coverage).

**Methods:** This study is a retrospective cohort design using TB reporting and treatment management data in South Korea. We analyzed PPM coverage through multilevel logistic regression and Empirical Bayesian (EB) estimation according to individual and area-level variables and their interaction.

**Results**: Patients aged 0-29 years, women, of Korean nationality, treated at a general hospital, a one-time reporting, urban areas, and the lowest deprivation index (DI) showed higher PPM coverage. Due to the cross-level interaction, PPM coverage in the urban areas (slope=-0.048, p<0.001) had a higher level but a steeper negative deprivation gradient than in rural areas (slope=-0.015, p<0.001). For a general hospital, the PPM coverage in urban is high but more significantly decreased than in rural areas with the higher DI (Urban: slope=-0.047, p < 0.001, Rural: slope=-0.031, p < 0.001). For clinics and hospitals, the effect of DI did not appear in urban areas, but in rural areas, the higher the DI, the higher the PPM coverage with a slope of 0.046 (p<0.001) and 0.063 (p<0.001), respectively.

**Conclusions:** The PPM program created a significant disparity in PPM coverage between urban-rural areas and type of healthcare provider (HCP) according to deprivation. Considering the high risk of TB incidence in areas with higher deprivation, institutional improvement and program redesign are needed to improve accessibility and equity.

**Keywords**

National Tuberculosis Program, health disparities, social determinants, accessibility, equity

**Introduction**

한국은 결핵(TB)을 주요 공중 보건 문제로 간주하고 2006년 결핵퇴치계획을 수립하였고, 2013년부터는 5년 단위 국가결핵 전략계획을 수립하고 있다. 세계보건기구는 PPM(Public-Private Mix) 전략을 DOTS(Directly observed treatment, short-course)를 비롯한 필수 결핵 치료서비스(promote access to quality TB care)의 적용을 확대하기 위한 핵심수단으로 활용하고 있다. 2001년부터 전 세계적으로 민간 부문 참여를 늘여 전반적인 결핵 관리 역량을 확대하였다. 한국 정부도 이에 영향을 받아 2009년부터 연간 250건 이상의 결핵을 치료하는 종합병원 22개를 대상으로 결핵 전문 간호사(TB-specialty nurse)를 파견하여 결핵환자에 대한 복약감독 등 결핵치료지원을 제공하는PPM 시범사업을 운영하였다. 2011년에는 PPM 사업을 전국으로 확대하여 252개 보건소와 100건 이상의 결핵을 치료하는120개의 의료기관이 참여하였다 [1-4]. 보건소가 지역 결핵관리 당국의 역할을 수행함과 동시에 결핵치료의 일차의료기관 역할을 하고 있으나, 치료환자수가 많은 대형 종합병원 위주의 “사업참여기관에 의뢰”하는 체계로 운영되고 있다. 이것은 치료지원의 접근성을 중요하게 고려하는 세계보건기구의 “모든 의료기관의 참여 전략”과 차이가 있다.

모든 의료 제공자를 참여시키는 것을 목적으로 하는 PPM 접근법은 4가지(결핵  사례 발견 증가, 결핵 치료 결과 개선, 접근성과 형평성 향상, 환자의 재정적 부담 감소)의 목표를 가지고 있다 [5]. 한국의 PPM 사업과 관련한 결핵 치료 결과 개선에 관한 연구는 시행되었으나 [4, 6], 접근성과 형평성에 대한 연구는 아직 시행되지 않았다. 결핵은 국가 간, 지역 간에 큰 사회적 격차가 있으며, 가장 가난한 사람들에게 가장 높은 위험을 갖는다는 것이 잘 알려져 있다 [7]. 높은 발생 위험을 가진 지역이 치료에서도 열악한 접근성과 품질을 가질 경우, 격차는 더 커질 수밖에 없다. 따라서 사업을 체계적으로 평가하고 개선 전략을 제시하기 위해서는 개인 요인과 지역관련 요인을 동시에 고려한 접근성과 품질의 차이 여부와 그 영향요인에 대한 연구가 필요하다.

결핵의 예방과 관리에서 지역은 주요한 요인이다. 전통적 생태학적 분석, GIS(Geographic Information System )를 활용한 연구를 통해 지역의 박탈이 원인이라는 근거가 있고, 사회적 위험요인(열악한 주거 환경, 높은 실업률, 지역 이민자 비율, 낮은 가계소득, 높은 불평등 및 빈곤의 복합지수 등)과 결핵 발생과의 관련성이 고소득 및 저소득 국가에서 시행한 연구에서 확인되고 있다 [8]. 다수준 분석은 개인과 지역 수준 요인의 영향 여부와 교호작용을 파악할 수 있어서, 감염병 연구에서도 중요성이 크다 [9]. 결핵 발생과 신고 [8, 10], 전파 [11], 진단과 치료에 대한 지침준수 [12]와 공간적 분포 특성 [13]과 다제내성 결핵의 분포 [14]에 관한 다수준 분석 연구가 시행되었다. 지역은 물질적 기반 시설과 집합적 사회적 기능을 통해 거주자들에 의한 구성적 요인과 별개로 건강에 영향을 미칠 수 있으므로 적절하게 운영화(operationalize)하면 지역과 건강 결과를 연결하는 인과 관계 가설을 검증할 수 있다. 지역의 물질적 및 집합적 사회적 기능은 의료기관 분포와 결핵 치료기관의 접근성을 결정하고, 지역 보건소의 결핵관리프로그램과 같은 공중보건기능은 지역의 집합적 사회적 기능으로 치료 지원에 영향을 준다 [15].

본 연구의 목적은 다수준 분석을 시행하여 PPM 사업의 주요한 평가 지표인 치료지원 가능성을 접근성과 형평성 관점에서 검토하는 것이다.

**MATERIALS AND METHODS**

**연구대상자**

한국의 결핵 신고와 치료 관리 데이터베이스를 사용한 후향적 코호트 연구로, 2017년 7월 질병관리본부로부터 2012년부터 2016년까지의 결핵 신환자에 대한 자료를 제공받았다. 결핵 신고 자료에는 지역보건소에서 확인한 환자가 실제 거주하는 주소지가 포함되어 있어 이를 활용하여 지역수준 변수와 연결하였고, 치료 관리 자료에는 치료를 제공한 의료기관의 종류가 기록되어 있다. 결핵 치료기간은 최소 6개월로 2016년 신고환자의 최종 치료결과 확인이 불완전하여 제외하고 2012년에서 2015년 사이에 신고된 신환자로 코호트를 구성하였다. 이 중 리팜핀 내성(RR) 또는 다제내성(MDR) 환자 1,810명은 제외하였고, 다른 의료 시설에서 두 번 이상 신고한 총 19,308명의 환자는 단일 환자로 포함하였다. 주요 변수에 결측치가 있는 36명을 제외한 137,865명의 약제 감수성 결핵 신환자 코호트 (drug-susceptible new TB patients cohort)가 연구대상이다.

**변수정의와 자료 처리**

결과변수인 개인별 결핵 치료 지원(PPM coverage)은 신고 후 결핵치료기관 안내를 받아 환자가 선택하고 등록한 치료 의료기관이 결핵전문간호사(TB specialty nurse)가 복약 여부를 감독하는 보건소 또는 PPM협력 의료기관인 경우로 정의하였다.

개인수준의 설명변수는 연도(2012, 2013, 2014, 2015), 연령군(0~29, 30~64, ≥65), 성별, 국적(Korean, Immigrant), 치료의료기관 종류(보건소를 포함한 의원, 종합병원, 병원), 결핵의 유형(폐외결핵, 도말양성 폐결핵, 도말음성 폐결핵), 신고된 횟수(1, 2, ≥3)를 포함하였다. 의원과 보건소는 특성이 다르지만, 결핵환자가 선택할 수 있는 상대적으로 접근성이 높은 일차의료기관으로 기능하는 점을 고려하여 분석 시 통합하였다. 의원급 의료기관 중 PPM 협력에 참여하는 기관에는 도시지역에 위치한 일부 대한결핵협회 부설 의원이 있다. 지역수준은 개인의 거주지에 따라 지역(district)(시,군,구)으로 구분하였다. 이 지역단위는 자치권한이 있으며 공식적인 통계자료가 제공되는 최소행정단위이다(보건소는 252개소이지만 행정구역 변동을 고려하고, 자치권이 있는 지역만 포함하여 245개소를 대상으로 함).

지역 수준의 설명변수는 박탈지수(Deprivation Index, DI)와 도시화정도를 사용하였다. 도시는 대도시 지역의 군구와 도지역의 시를 포함하였고, 그 이외의 지역은 농촌으로 분류하였다.

박탈지수는 다른 연구에서 활용한 방법 [16, 17]을 활용하였고 영향 지연시간(lag time)을 고려하여2010년 인구주택총조사의 표본을 활용하여 거주자의 주택환경, 학력, 가구주의 사회계층, 고령자 비율, 1인가구, 차가 없는 가구, 비아파트, 여성가구, 이혼, 별거 가정 등을 포함하여 산출하였고 지수 값이 높을수록 박탈수준이 높은 것을 의미한다. 기술 분석에서는 분포와 결과변수와의 단순 관련성을 살펴보기 위해 5분위로 구분하여 제시하였고, 선형성이 확인되어 다수준분석에서는 중앙화한 연속변수로 분석에 포함하였다.

**통계 분석**

결핵 치료 지원(PPM coverage)에 대한 다수준 로지스틱 회귀분석은 단순 모형에서 복잡한 모형으로 확장하여 모형을 구축하였다. 우선 지역 차원의 변이를 확인하기 위해 변량 절편(random intercept)만 포함한 “빈(empty)” 모형을 추정하고, 이후 개인 특성을 모형에 포함시켜 지역의 변이가 개인 구성 수준의 차이로 설명되는 정도를 조사하였다. 산출된 오즈비는 기준 범주에 대한 해당 범주의 오즈비로 1보다 큰 값일 경우 해당 범주의 결핵 치료 지원을 받을 가능성의 증가를 의미한다. 다음으로 지역 변이가 지역 특성에 의해 조건화 되었는지 여부를 조사하기 위해 지역박탈지수, 도시화 수준을 추가하였고, 치료의료기관 종류와 교호작용을 포함하여 지역수준 변이의 설명정도를 살펴보았다 [18, 19]. 본 연구의 결과변수는 이분형 반응변수로서 급내상관계수(ICC, intraclass correlation)와 같은 연속형 변수에 적용하는 분산분할계수는 제한점이 있다. 따라서 개인 변수의 오즈비(odds ratio)와 지역 수준의 변이를 직접 비교하기 위해 MOR(median odds ratio)과 IOR-80(Interval Odds ratio 80%) 값을 제시하였다. MOR은 집단 이질성(group heterogeneity)(군집 효과, cluster effect)을 정량화한 값이며, IOR-80은 지역 수준 고정 효과(area level fixed effect)와 변량 잔차 변이(random residual variations)를 고려하여 지역수준 공변량(covariate) 효과를 정량화 하는 값이다 [20, 21]. 지역수준별 박탈지수와 도시화 정도 간의 복잡한 관련성을 시각적으로 나타내기 위해 분석 시 활용한 모형(Model 2 and model 3)에 경험적 베이지안 추정법을 적용하여 산출한 지역별 추정치를 그래프로 제시하였다 [18]. 경험적 베이지안 추정법은 집단 별 자료뿐만 아니라 다른 집단과 공유하는 특성을 함께 고려할 수 있는 장점이 있어서 활용이 증가하고 있다 [22]. 개인 수준의 특성을 구체적으로 고려한 지역별 추정치를 산출하기 위해 모형 3에 적용할 때 연도, 연령군, 성별, 결핵유형, 국적, 신고횟수를 각각 2015년, 30~64세, 여성, 도망양성 폐결핵, 한국국적, 1회 신고사례로 값을 지정한 후 도시화정도와 박탈지수별로 지역 변량 효과(random effect)를 포함하여 지역과 치료기관별로 유형별 가장 대상자가 많은 집단의 최근 확률을 산출하였다(다른 연령, 성별, 국적에 대한 결과는 부록에 제시함). 도시화 정도와 박탈지수만을 고려한 모형 2는 모형 3과 같은 개인변수 값과 가장 대상자가 많은 종합병원으로 치료의료기관을 지정하고 지역 변량 효과(random effect)를 포함하여 지역 확률을 산출하였다. PPM협력기관이 치료의료기관일 사건분율이 상대적으로 높아 오즈비가 과다추정될 수 있어 그 해석이 제한될 수 있는 점을 고려하고, 역학적 교호작용을 평가하는데 승수 모형보다는 가산 모형이 더 현실을 반영할 수 있다는 근거 [23]에 따라 지역별 환자 규모의 영향을 반영하기 위해 지역 추정치에 해당집단의 지역별 환자수로 가중치를 부여한 가중 회귀분석을 시행하여 도시화 정도별로 박탈지수와 결핵치료 지원의 관련성(figure 1-(1))과 치료의료기관과의 교호작용을 고려한 박탈지수와 결핵치료 지원과의 관련성(figure1-(2)을 회귀계수로 제시하였다.

Figure-2는 지역별로 PPM 협력의료기관을 이용할 수 없거나, 보건소를 치료받기를 꺼리는 이유 등으로 치료 지원을 받지 않은 환자가 이용하는 의료기관의 지역별 상황을 살펴보기 위해, 지역별로 치료지원을 받지 않은 환자수와 이들이 이용한 치료기관 수의 비를 산출하여 지역별 특성에 따라서 제시하였다. 앞의 그림과 마찬가지로 해당 지역별 대상자수에 가중치를 부여한 회귀분석을 시행하여 제시하였다.

**참여자 동의와 연구 윤리 승인**

연구계획서는 부산대학교병원 기관윤리심의위원회(IRB Approval Number H-1708-030-058)에서 심의하였다. 설명과 동의서작성(informed consent)에 대한 의무는 통상적으로 수집되는 자료로 수행된 후향적 코호트 연구로 인정받아 면제되었다. 자료는 사전에 익명화 처리하여 분석하였다.

**Results**

**연구 대상자의 특성**

연구에 포함된 137,865명은 연도별로 가장 이전시기(2012년, 27.7%), 30~64세 연령군(48.9%), 남성(57.3%), 한국 국적(96.1%)이 가장 높은 분율을 차지하였다. 79.1%는 폐결핵이었고 20.9%는 폐외 결핵 환자였다. 치료시설 유형별로는 종합병원(77.1%), 신고횟수별로는 1회 신고자(86.0%), 거주지역별로는 도시지역(87.2%)의 분율이 가장 높았다.

**결핵 치료 지원**

전체 결핵 치료지원 확률(PPM coverage)은 76.0%(95%CI, 75.8-76.2)였다. 2012~2016년까지의 기간동안 년도별로 통계적 유의한 차이와 경향성은 나타나지 않았다. 개인수준 변수별로는 0-29세(79.2%), 여성(77.3%), 한국 국적(76.1%), 종합병원에서 치료받은 경우(84%), 1회신고자(76.7%)에서 통계적으로 유의한 수준으로 높은 양상을 보였다. 지역수준 변수에서는 도시거주자(77.1%), 박탈지수가 가장 낮은 5분위(80.7%)에서 가장 높은 양상이었다(Table 1).

표2에는 다수준 로지스틱 회귀분석에서 추정된 고정(fixed) 및 변량(random) 매개변수(parameter)를 제시하였다. 빈(empty)모형( Model 0)에서 개인, 지역, 교호작용을 포함한 모형(Model 3)까지 치료지원에 대한MOR값의 신뢰구간에 1을 포함하지 않아 지역별로 유의한 차이가 나타났지만 집단의 이질성(군집효과)은 1.004로 크지 않았다. 개인 및 지역 수준의 변수를 포함한 경우에도 집단의 이질성 수준은 크게 변하지 않았다. 개인 수준의 변수를 추가한 경우(Model 1)는 Table 1과 동일하게 년도를 제외한 모든 변수에서 통계적으로 유의한 관련성이 나타났고, 지역간 변이는 20% 감소하였는데 이는 각 지역의 개인수준 변수 구성의 차이가 지역간 변이의 20%를 설명하는 것을 의미한다. 박탈지수와 도시화 수준을 포함한 경우(Model 2)에서 지역간 변이는 소폭 감소(-1.4%)하였지만 이전 모형과 유사한 수준의 군집효과가 나타났다(표에 제시되지 않았지만 도시화 수준을 포함한 경우 4.8%의 변이가 증가하였고, 박탈지수를 포함한 경우 -5.9% 감소함). 지역수준 변수의 고정효과는 도시지역일수록, 박탈수준이 높을 수록 치료 지원 오즈비가 감소하였다. IOR-80으로 도시화와 박탈지수의 효과를 살펴보면, 두 변수 모두 IOR-80의 구간 값에 1을 포함하지 않았고 구간도 넓지 않았는데 이는 치료 치원 오즈비의 군집효과가 크지 않았지만 상당 수준을 도시화정도와 박탈지수로 설명한다는 것을 의미한다.

모형 2에서는 도시화정도와 박탈지수 사이의 2원 교호작용이 확인되었고 모형 3에서는 도시화정도, 박탈지수와 치료의료기관선택 사이에서 3원(three-way) 교차수준 교호작용 효과가 확인되었다. 그 결과는 표3으로 정리하여 제시하였다. 모형 2로부터 산출한 교호작용 효과는 도시지역의 치료지원 가능성이 낮았고(OR : 0.93), 박탈지수가 높을수록 농촌지역은 치료지원 가능성이 높아졌고(OR=1.15), 도시지역은 낮아지는 효과(OR=0.84)가 확인되었다. 모형 3로부터 산출한 교호작용 효과는 같은 병원을 이용하더라도 도시지역이 농촌 지역에 비해 치료지원을 받을 가능성이 높았고(OR: Hospital Urban/Hospital Rural=2.67), 병원에 비해 종합병원과 의원(보건소포함)을 이용할 경우 결핵치료지원을 받을 가능성이 크게 증가하였다. 그러나 그 수준은 농촌 지역(OR : Clinic Rural/Hospital Rural=46.09, General Hospital Rural /Hospital Rural=66.37)보다 도시 지역(OR : Clinic Urban/Hospital Rural=30.76, General Hospital Urban /Hospital Rural=57.61)이 낮았다. 박탈지수 단위 값 변화를 나타내는 기울기도 치료의료기관과 도시화 정도에 따라 다르게 산출되었다. 종합병원의 경우 박탈수준이 높은 지역에 거주할 경우 감소하였는데 그 수준은 도시지역(OR=0.77)이 농촌지역(OR=0.98)에 비해 컸다. 의원과 병원의 경우 박탈수준이 높은 지역에 거주할수록 증가하였는데 그 수준은 농촌 병원(OR=2.30), 농촌 의원(OR=1.68), 도시 병원(OR=1.16), 도시 의원(OR=1.12)의 순서였다.

그림 1-a는 모형2를 적용하여 경험적 베이지안 추정법으로 지역별 추정치를 산출하여 TB 치료 지원 확률에 대한 도시화정도와 박탈수준의 효과를 나타낸 것이다. 박탈지수 분포에서 농촌지역이 더 큰 값에 분포하였고, 박탈지수가 증가할수록 지역별 결핵 치료 지원 확률이 감소하는 경향이었다. 다수준 로지스틱 회귀분석에서 산출된 승산 척도(multiplicative scale)에는 농촌 지역의 박탈수준에 따른 치료지원 가능성이 증가하는 것으로 나타났지만, 소지역 추정 후 산출한 가산 척도(additive scale)의 기울기에서는 감소하는 것으로 산출되었다. 승산 척도에서 양이던 값이 가산 척도에서 음의 값으로 나타는 농촌지역(기울기-0.015, p<0.001) 보다는 두 척도 모두에서 음의 값을 나타낸 도시지역(기울기-0.048, p<0.001)에서 감소의 기울기가 더 컸다.

그림 1-b는 모형 3을 적용하여 같은 추정법으로 산출한 것으로 결핵 치료 지원 확률에 대한 도시화정도와 박탈수준, 치료의료기관의 3원(three-way) 교차수준 교호작용 효과를 시각적으로 보여준다. 도시지역의 경우 박탈지수가 작은 값에 분포하여 치료받을 때 결핵 치료 지원 확률이 높지만, 박탈지수가 클수록 종합병원 치료 시 결핵 치료 지원 확률이 농촌지역보다 51% 더 감소하였다(Urban: slope=-0.047, p<0.001, Rural: slope=-0.031, p<0.001). 승산 척도에서 작은 증가 양상을 보였던 의원과 병원급에 대한 박탈지수의 영향은 도시지역에서는 나타나지 않았으나, 승산 척도에서 큰 증가 양상을 보였던 농촌지역은 박탈지수가 큰 지역에서 의원급과 병원급에서 치료를 받을 경우 각각 0.046(p<0.001), 0.063(p<0.001)의 기울기로 결핵치료지원을 받을 확률이 증가하여 종합병원의 치료지원이 감소한 것을 보완하였다. 그럼에도 불구하고, 농촌 지역은 치료지원 확률이 도시 지역보다 낮았다.

Figure2는 지역별로 치료 지원을 받지 않은 환자수와 이들이 이용한 치료기관 수의 비(ratio)를 도시화정도와 박탈수준에 따라 나타낸 것이다. 도시지역이 치료 지원을 받지 않은 환자가 많았지만, 비(ratio)의 값(농촌지역 median=0.09, 도시지역 median=0.06)과 박탈수준에 따른 기울기(농촌지역slope=0.020, p<0.001, 도시지역slope=0.005, p<0.001)는 농촌지역이 컸다. 이는 한국의 PPM 협력체계가 치료건수가 많은 병원 기반으로 운영되어 농촌 지역에 가용한 PPM 협력 의료기관이 부족한 상황을 만들었고, 이로 인해 농촌 지역의 거주자들은 도시 지역의 PPM 협력 기관에서 치료받기 어려운 경우 PPM 협력기관이 아닌 의료기관에서 치료를 받을 수 밖에 없는 상황이며, 그 수준은 지역 박탈수준과 비례적으로 관련되어 있음을 의미한다. 이는 결핵 관리 정책이 결핵치료서비스의 수준과 치료지원 모두에서 도시-농촌, 지역박탈 수준에 따른 격차를 만들어 내고 있는 것으로 해석될 수 있다.

**Discussion**

본 연구는 다수준 분석을 활용하여 사업 초기인 2012~2015년 한국의 120개 병원과 252개 보건소로 이루어진 PPM 협력 결핵 치료 지원 체계(2009년 22개소, 2011년 전국적 사업 확대로 120개소 [1], 2015년 127개소, 2022년 현재 184개소 [24]로 참여 병원은 다소 증가하였지만, 도시지역 병원위주의 지원체계 유지)의 운영 성과를 접근성과 형평성 측면에서 평가하여 성과와 한계를 확인하였다. 이 결과를 활용하면 국가 결핵관리의 주요지표인 치료결과(성공, 실패, 사망 등)에 대한 개인과 지역 수준 요인의 인과 관계 경로와 기전을 파악할 수 있게 하여 결핵퇴치를 위한 지역 기반 전략 개발에 활용할 수 있다.

**공중보건 함의**

한국의 결핵치료지원체계는 참여기관의 수가 작을 뿐만 아니라, 2015년까지 67%(252개 중 80개) 지역에만 협력기관이 소재하고(대부분 도시 지역), 결핵환자를 치료한 종합병원 365개소 중 133개소(36.4%), 병원 1395개소 중 4개소(0.3%), 의원 1189개소 중 2개소(0.2%)만 PPM 협력사업에 참여하여 지역 보건소가 100% 사업에 참여하여 의뢰와 직접 지원을 하더라도 적절하고 형평한 치료지원을 제공하기에 어려운 상황이다. 본 연구 결과를 적용하면 집단별로 여성 30~64세 연령군 62.9~86.6%, 남성 65세 이상 연령군 55.7~82.1%, 이주민 52.6~80.9%(Table S1)의 분포를 가지는데, 지역 격차와 인구 집단 특성이 교차할 때 50% 수준만 지원만 가능하여 접근성의 문제는 심각하다. 특히 노인, 이주민, 박탈 수준의 사회적 맥락을 고려하면 형평성 차원의 문제도 크다.

전국적 PPM 사업 도입 후 민간 부문의 치료성공률이 5년간 70.3%에서 83.9%로 향상된 것으로 나타났지만 [4] 이는 치료성공률이 높은, 사업에 참여한 민간 대형 의료기관으로 더 많은 환자가 의뢰된 효과일 가능성이 높다. 중앙과 지역 보건당국(PPM사업 포함)이 민간의료기관에게 결핵 치료 지침을 따르도록 규제하고 유인하거나, 공중보건 기능과 연결시킬 수 있는 체계가 갖추어지지 않은 상황에서 자발적으로 전반적 성과 향상이 있었을 것이라고 기대할 근거가 부족하다. 이런 문제를 해결하기 위해서는 “사업참여기관에 의뢰하는 전략”에서 WHO가 권고하는 결핵 환자들이 이용하는 모든 의료기관들을 PPM 협력 체계에 포함하여 지원하는 “모든 의료기관의 참여” 전략으로의 전환이 필요하다. 현재의 사업구조로 계속 운영된다면 PPM 치료지원을 충분히 받지 못하는 지역과 인구집단은 (치료성공률OR [6] PPM종합병원 1, Non-PPM 종합병원 0.66, PPM 병원 0.64, non-PPM 병원 0.49, clinic 0.39 적용 시) 치료성공률도 매우 낮을 수 밖에 없다.

강력한 관리의 분권(intensive decentralization of care)을 통해 DOTS(단기 직접 관찰 치료) 전략을 환자 가까이에서 지원했을 때 이동 및 기회 비용이 최소화되어 환자의 순응도가 훨씬 높아졌고 [25], PPM 협력기관에 가난한 지역의 기관이 포함되거나 서비스를 제공하는 프로그램이 마련되었을 때 접근의 형평성을 높일 수 있었다는 결과 [26]는 “모든 의료기관을 참여” 전략의 중요한 근거이다. 이들 정책과 함께 지역 자료를 활용하기 위한 지원 정책 [27]이나, 권역 단위로 해당지역 내 과정, 결과 지표를 체계적으로 모니터링 할 수 있는 코호트 리뷰와 같은 지역별 관리 정책[10-13]을 도입하는 것이 고려되어야 한다. 새로운 도전에 필요한 정책적 보완이 이루어 지지 않을 경우, PPM협력사업의 정책은 의도하지 않은 건강 불평등 확대로 이어질 수 있을 것이다.  [28, 29]

**방법론적 문제**

본 연구는 방법론적 관점에서 몇 가지 강점이 있다. 첫째, 개인별 치료지원여부를 결과변수로 사용하여, 지역별 인구집단별 결핵치료시설 여부나 수를 지표로 단순하게 활용하지 않고 보편적 건강 및 결핵 치료 보장의 관점에서 접근성과 형평성을 심층적으로 평가한 최초의 연구이다[1–3]. 둘째 결핵치료지원을 위한 PPM 사업을 전면 도입한 후 사업이 안정화된 시기의 4년간의 결핵등록 환자를 대상으로 분석을 시행하여 큰 표본 수와 대표성을 확보하였다. 셋째, 다수준 모형으로 개인수준과 지역 수준의 변수를 동시에 고려하여 치료 지원 확률에서 각각의 변수들이 지역 수준 변이를 설명하는 정도를 확인하였고, 치료기관 종류와 지역 특성 사이의 교호작용을 확인하였다. 이는 지역별 구분과 도시화정도와 박탈수준의 효과가 병원의 여부에 영향을 미치는 조건화된 선택이므로 지리정보 시스템을 직접 사용하지 않았지만 PPM협력의 치료 지원의 접근성의 효과를 나타낼 수 있다. 지역별로 구체적으로 정량화된 접근성 지표는 개인별 거주지와 치료 병의원의 소재지에 대한 정보를 활용한 후속 연구에서 산출될 수 있을 것이다.

몇 가지 제한점도 존재한다. 첫째, 한국의 결핵 신고체계는 WHO 지침에서 포함하지 않은 개인의 사회경제적 수준에 대한 변수를 수집하지 않고 있어서[5] 본 연구에서도 분석에 활용할 수 없었다. 모형에 개인의 사회경제적 지위의 구성 효과가 고려되지 못하여 잔여 혼란(residual confounding)의 가능성을 배제할 수 없다. 그러나 잔여 혼란은 효과의 크기에만 영향을 줄 가능성이 크므로 본 연구 결과의 전반적인 해석에 큰 영향을 주지는 않을 것이다. 둘째, 지역(구군) 단위의 지역 구분에 따른 변이 정도가 통계적으로 유의한 수준이었지만 크지 않았다. 그러나, 행정구역을 활용한 지역구분이 설명하는 변이정도로 모형의 적절성 여부를 판단하는 것을 권장하지 않고 [19, 30], 의료기관 중 소수의 기관만이 PPM 협력 체계에 참여하는 상황에서 보건당국의 활동이 치료기관 의뢰하거나 직접 치료를 제공하는 등 선택에 영향을 주고 있으므로, 정책으로 형성되는 집합적 사회적 속성을 반영하는 지역 단위로 고려할 수 있다. 후속 연구를 통해 PPM 의료기관의 진료권과 지역내 의료기관의 존재 여부 등을 추가로 포함해 교차 분류된 구조(cross-classified structure )에 대한 분석 연구를 시행하여 그 효과의 상대적 중요성과 크기를 확인할 필요가 있다 [31].

**Conclusion**

2011년부터 전국적으로 확대 시행한 PPM 협력사업은 치료 환자수(case load)가 많은 병원 기반 PPM 협력 사업과 지역 보건소의 제한적 치료지원은 전반적으로 치료지원 수준을 높이는 효과(76%)가 있었지만, 도시화수준, 박탈 수준과 치료지원기관 선택의 3원 교차수준 교호작용의 결과로 지역간 결핵 치료 지원(PPM coverage)에 지역박탈지수에 따른 큰 기울기를 가진 격차를 만들었다(도시지역 기울기-0.048, p<0.001 농촌지역 기울기-0.015, p<0.001). 지역박탈수준이 큰 지역의 높은 결핵 발생 위험을 고려하면 효율성과 함께 형평성 수준을 향상시킬 수 있는 제도적 개선과 프로그램의 보완이 필요하다.

**Reference**

1.Cho KS. Tuberculosis control in the Republic of Korea. Epidemiology and Health 2018;40:e2018036.

2.Go U, Park M, Kim U-N, Lee S, Han S, Lee J, et al. Tuberculosis prevention and care in Korea: Evolution of policy and practice. Journal of Clinical Tuberculosis and Other Mycobacterial Diseases 2018;11

3.Kim J, Yim J-J. Achievements in and Challenges of Tuberculosis Control in South Korea. Emerging Infectious Diseases 2015;21:1913-1920.

4.Yu S, Sohn H, Kim H-Y, Kim H, Oh K-H, Kim H-J, et al. Evaluating the impact of the nationwide public–private mix (PPM) program for tuberculosis under National Health Insurance in South Korea: A difference in differences analysis. PLoS Medicine 2021;18:e1003717.

5.Uplekar M, Lönnroth K. Engaging all health care providers in TB control : guidance on implementing public-private mix approaches. WHO IRIS 2006

6.Son HJ, Mok JH, Lee MY, Park WS, Kim SJ, Lee JS, et al. Status and Determinants of Treatment Outcomes Among New Tuberculosis Patients in South Korea: A Retrospective Cohort Study. Asia Pacific Journal of Public Health 2021;33:907-913.

7.Lönnroth K, Jaramillo E, Williams BG, Dye C, Raviglione M. Drivers of tuberculosis epidemics: The role of risk factors and social determinants. Social Science & Medicine 2009;68:2240-2246.

8.Ximenes ARAd, Albuquerque FMdd, Souza WV, Montarroyos UR, Diniz GTN, Luna CF, et al. Is it better to be rich in a poor area or poor in a rich area? A multilevel analysis of a case-control study of social determinants of tuberculosis. 2009;38

9.Roux AVD. A glossary for multilevel analysis. Journal of Epidemiology and Community Health 2002;56:588.

10.Cramm JM, Koolman X, Møller V, Nieboer AP. Socio-economic status and self-reported tuberculosis: a multilevel analysis in a low-income township in the Eastern Cape, South Africa. Journal of Public Health in Africa 2011;2:e34.

11.Oren E, Narita M, Nolan C, Mayer J. Neighborhood socioeconomic position and tuberculosis transmission: a retrospective cohort study. BMC Infectious Diseases 2014;14:227.

12.Thongraung W, Chongsuvivatwong V, Pungrassamee P. Multilevel factors affecting tuberculosis diagnosis and initial treatment. Journal of Evaluation in Clinical Practice 2008;14:378-384.

13.Im C, Kim Y. Spatially Filtered Multilevel Analysis on Spatial Inequality of Tuberculosis in Gyeongsangbuk-do, Korea. Journal of Health Informatics and Statistics 2021;46:88-99.

14.Arroyo LH, Yamamura M, Ramos ACV, Campoy LT, Crispim JdA, Berra TZ, et al. Determinants of multidrug‐resistant tuberculosis in São Paulo—Brazil: a multilevel Bayesian analysis of factors associated with individual, community and access to health services. Tropical Medicine & International Health 2020;25:839-849.

15.Macintyre S, Ellaway A, Cummins S. Place effects on health: how can we conceptualise, operationalise and measure them? Social Science & Medicine 2002;55:125-139.

16.Global Burden of Tuberculosis.

17.Kim KH, Chun J-H, Sohn HS. The influence of regional deprivation index on personal happiness using multilevel analysis. Epidemiol Health 2015;37:e2015019-2015010.

18.Diez-Roux AV. Multilevel Analysis in Public Health Research. Annual Review of Public Health 2000;21:171-192.

19.Duncan C, Jones K, Moon G. Context, composition and heterogeneity: Using multilevel models in health research. Social Science & Medicine 1998;46:97-117.

20.Merlo J, Chaix B, Ohlsson H, Beckman A, Johnell K, Hjerpe P, et al. A brief conceptual tutorial of multilevel analysis in social epidemiology: using measures of clustering in multilevel logistic regression to investigate contextual phenomena. Journal of Epidemiology and Community Health 2006;60:290-297.

21.Larsen K, Merlo J. Appropriate Assessment of Neighborhood Effects on Individual Health: Integrating Random and Fixed Effects in Multilevel Logistic Regression. American Journal of Epidemiology 2005;161:81-88.

22.Rao JNK, Molina I. Small area estimation: John Wiley & Sons; 2015

23.Rothman KJ. Epidemiology: an introduction: Oxford university press; 2012

24.KCDC. National tuberculosis control program guideline 2022. 2022:1-669.

25.WHO. Guidelines for treatment of drug-susceptible tuberculosis and patient care. 2017 update ed. Geneva: World Health Organization; 2017

26.Malmborg R, Mann G, Squire SB. A systematic assessment of the concept and practice of public-private mix for tuberculosis care and control. International Journal for Equity in Health 2011;10:49.

27.Theron G, Jenkins HE, Cobelens F, Abubakar I, Khan AJ, Cohen T, et al. Data for action: collection and use of local data to end tuberculosis. The Lancet 2015;386:2324-2333.

28.Benach J, Malmusi D, Yasui Y, Martínez JM, Muntaner C. Beyond Rose's Strategies: A Typology of Scenarios of Policy Impact on Population Health and Health Inequalities. International Journal of Health Services 2011;41:1-9.

29.Frohlich KL, Potvin L. Transcending the Known in Public Health Practice. American Journal of Public Health 2011;98:216-221.

30.Merlo J, Chaix B, Yang M, Lynch J, Råstam L. A brief conceptual tutorial on multilevel analysis in social epidemiology: interpreting neighbourhood differences and the effect of neighbourhood characteristics on individual health. Journal of epidemiology and community health 2005;59:1022-1028.

31.Goldstein H. Multilevel Cross-Classified Models. Sociological Methods & Research 1994;22:364-375.

Table 1. Treatment support rates of Tuberculosis Patients According to Study Characteristics

|  |  | Study Population | | Rates of TB Treatment support | |
| --- | --- | --- | --- | --- | --- |
|  |  | Number (%) | P-value | Rate (95% CI) | P-value |
| Total |  | 137,865(100.0) |  | 76.0 (75.8-76.2) |  |
| Individual variable | |  |  |  |  |
| year |  |  | <0.001 |  | 0.153 |
|  | 2012 | 38,138 (27.7) |  | 75.6 (75.2-76.1) |  |
|  | 2013 | 35,156 (25.5) |  | 76.1 (75.7-76.6) |  |
|  | 2014 | 33,877 (24.6) |  | 76.3 (75.9-76.8) |  |
|  | 2015 | 30,694 (22.3) |  | 75.9 (75.4-76.4) |  |
| Age group |  |  | <0.001 |  | <0.001 |
|  | 0~29 | 22,836 (16.6) |  | 79.2 (78.7-79.7) |  |
|  | 30~64 | 67,466 (48.9) |  | 77.3 (77-77.6) |  |
|  | ≥ 65 | 47,563 (34.5) |  | 72.6 (72.2-73) |  |
| Gender |  |  | <0.001 |  | <0.001 |
|  | Men | 79,016 (57.3) |  | 75.0 (74.7-75.3) |  |
|  | Women | 58,849 (42.7) |  | 77.3 (77-77.6) |  |
| Nationality | |  | <0.001 |  | <0.001 |
|  | Korean | 132,431 (96.1) |  | 76.1 (75.8-76.3) |  |
|  | Migrant | 5,434 (3.9) |  | 73.8 (72.6-75.0) |  |
| Type of health care facility | |  | <0.001 |  | <0.001 |
|  | Clinic | 17,817 (12.9) |  | 73.9 (73.2-74.5) |  |
|  | General Hospital | 106,350 (77.1) |  | 84.0 (83.7-84.2) |  |
|  | Hospital | 13,698 (9.9) |  | 16.8 (16.2-17.5) |  |
| Type of TB | |  | <0.001 |  | <0.001 |
|  | Extra-pulmonary TB | 28,829 (20.9) |  | 79.8 (79.3-80.3) |  |
|  | Pulmonary, smear-TB | 41,068 (29.8) |  | 74.4 (73.9-74.8) |  |
|  | Pulmonary, smear+TB | 67,968 (49.3) |  | 75.3 (75-75.7) |  |
| Number of times notified | |  | <0.001 |  | <0.001 |
|  | 1 | 118,497 (86.0) |  | 76.7 (76.4-76.9) |  |
|  | 2 | 16,275 (11.8) |  | 72.8 (72.1-73.5) |  |
|  | ≥3 | 3,093 (2.2) |  | 66.1 (64.4-67.8) |  |
| Regional variable | |  |  |  |  |
| Residential Area | |  | <0.001 |  | <0.001 |
|  | Rural | 17,622 (12.8) |  | 68.7 (68.0-69.4) |  |
|  | Urban | 120,243 (87.2) |  | 77.1 (76.8-77.3) |  |
| Deprivation index | |  | <0.001 |  | <0.001 |
|  | 1st(least) | 27,584 (20.0) |  | 80.7 (80.2-81.1) |  |
|  | 2nd | 27,878 (20.2) |  | 79.2 (78.8-79.7) |  |
|  | 3rd | 28,150 (20.4) |  | 75.3 (74.8-75.8) |  |
|  | 4th | 26,732 (19.4) |  | 76.4 (75.9-76.9) |  |
|  | 5th(most) | 27,521 (20.0) |  | 68.3 (67.7-68.8) |  |

Abbreviations: TB, tuberculosis

P values by $\chi^{2}$ test

Table 2. Parameter estimates from multilevel logistic regression model (OR, 95%CI)

|  | **Model 0** | | **Model 1** | | **Model 2** | | **Model 3** | |
| --- | --- | --- | --- | --- | --- | --- | --- | --- |
| **Characteristic** | **OR***^1^* | **95% CI***^1^* | **OR***^1^* | **95% CI***^1^* | **OR***^1^* | **95% CI***^1^* | **OR***^1^* | **95% CI***^1^* |
| **Fixed effect** |  |  |  |  |  |  |  |  |
| **Individual level** |  |  |  |  |  |  |  |  |
| Year |  |  |  |  |  |  |  |  |
| 2012 |  |  | (ref) |  |  |  |  |  |
| 2013 |  |  | 1.04 | (1.00, 1.08) | 1.03 | (1.00, 1.08) | 1.03 | (0.99, 1.07) |
| 2014 |  |  | 1.03 | (0.99, 1.07) | 1.03 | (0.99, 1.07) | 1.03 | (0.99, 1.07) |
| 2015 |  |  | 1.03 | (0.99, 1.08) | 1.03 | (0.99, 1.08) | 1.03 | (0.99, 1.07) |
| Age group(years) |  |  |  |  |  |  |  |  |
| 0~29 |  |  | (ref) |  |  |  |  |  |
| 30~64 |  |  | 0.90^***^ | (0.87, 0.94) | 0.91^***^ | (0.87, 0.94) | 0.90^***^ | (0.86, 0.93) |
| ≥ 65 |  |  | 0.75^***^ | (0.71, 0.78) | 0.75^***^ | (0.72, 0.78) | 0.74^***^ | (0.71, 0.77) |
| Gender |  |  |  |  |  |  |  |  |
| Men |  |  | (ref) |  |  |  |  |  |
| Women |  |  | 1.11^***^ | (1.08, 1.14) | 1.11^***^ | (1.08, 1.14) | 1.10^***^ | (1.07, 1.14) |
| Healthcare facility |  |  |  |  |  |  |  |  |
| Hospital |  |  | (ref) |  |  |  |  |  |
| Clinic |  |  | 13.20^***^ | (12.47,13.9) | 13.3^***^ | (1.95, 2.11) | 46.4^***^ | (35.4, 60.9) |
| General Hospital |  |  | 26.82^***^ | (25.53, 28.1) | 27.0^***^ | (25.7, 28.3) | 66.4^***^ | (52.4, 84.0) |
| Type of TB |  |  |  |  |  |  |  |  |
| Pulmonary, smear+TB | |  | (ref) |  |  |  |  |  |
| Extra-pulmonary TB |  |  | 0.97^***^ | (0.93, 1.01) | 0.97^***^ | (0.93, 1.02) | 1.10^***^ | (1.06, 1.15) |
| Pulmonary, smear-TB | |  | 0.90^***^ | (0.87, 0.94) | 0.90^***^ | (0.87, 0.94) | 1.08^***^ | (1.05, 1.12) |
| Nationality |  |  |  |  |  |  |  |  |
| Korean |  |  | (ref) |  |  |  |  |  |
| Migrant |  |  | 0.81^***^ | (0.75, 0.87) | 0.81^***^ | (0.75, 0.87) | 0.82^***^ | (0.77, 0.88) |
| Number of times notified |  |  |  |  |  |  |  |  |
| 1 |  |  | (ref) |  |  |  |  |  |
| 2 |  |  | 1.47^***^ | (1.41, 1.55) | 1.48^***^ | (1.41, 1.55) | 1.43^***^ | (1.37, 1.50) |
| ≥3 |  |  | 1.24^***^ | (1.13, 1.36) | 1.24^***^ | (1.12, 1.36) | 1.22^***^ | (1.10, 1.34) |
|  |  |  |  |  |  |  |  |  |
| **Area level** |  |  |  |  |  |  |  |  |
| Urbanicity |  |  |  |  |  |  |  |  |
| Rural |  |  |  |  | (ref) |  |  |  |
| Urban |  |  |  |  | 1.09^***^ | (1.04, 1.15) | 2.67^***^ | (2.11, 3.37) |
| Deprivation |  |  |  |  | 1.13^***^ | (1.08, 1.18) | 2.30^***^ | (1.96, 2.72) |
| Urbanicity × Deprivation |  |  |  |  |  |  |  |  |
| Urban × Deprivation |  |  |  |  | 0.74^***^ | (0.71,0.78) | 0.50^***^ | (0.42, 0.61) |
|  |  |  |  |  |  |  |  |  |
| **Cross- level** |  |  |  |  |  |  |  |  |
| Healthcare facility × Deprivation | |  |  |  |  |  |  |  |
| Clinic × Deprivation |  |  |  |  |  |  | 0.73^***^ | (0.59, 0.90) |
| General Hospital × Deprivation | | |  |  |  |  | 0.43^***^ | (0.36, 0.51) |
| Healthcare facility × Urbanicity | |  |  |  |  |  |  |  |
| Clinic × Urban |  |  |  |  |  |  | 0.25^***^ | (0.19, 0.33) |
| General Hospital × Urban | |  |  |  |  |  | 0.33^***^ | (0.25, 0.42) |
| Healthcare facility × Urbanicity × Deprivation | | |  |  |  |  |  |  |
| Clinic × Urban× Deprivation | | |  |  |  |  | 1.33^***^ | (1.04, 1.68) |
| General Hospital × Urban× Deprivation | | |  |  |  |  | 1.56^***^ | (1.28, 1.89) |
|  |  |  |  |  |  |  |  |  |
| **Random effect** |  |  |  |  |  |  |  |  |
| $\sigma$(95% CI) | 0.005(0.001,0.017) | | 0.004(0.001,0.017) | | 0.004(0.001,0.017) | | 0.004(0.001,0.017) | |
| MOR | 1.004(1.001-1.018) | | 1. 004 (1.001-1.016) | | 1. 004 (1.001-1.016) | | 1. 004 (1.001-1.016) | |

***: < 0.001Table 3. Effect of two-way and three-way interaction between healthcare facility, deprivation, and Urbanicity.

|  |  | Urbanicity | | Urbanicity × Deprivation | |
| --- | --- | --- | --- | --- | --- |
|  |  | aOR | IOR-80 | aOR | IOR-80 |
| model 2 | Rural | Ref) | - | 1.15(0.03) ^***^ | 1.15-1.17 |
|  | Urban | 0.93(0.03) ^***^ | 0.84-0.85 | 0.84(0.03) ^***^ | 0.84-0.85 |
|  |  | Healthcare facility × Urbanicity | | Healthcare facility × Urbanicity × Deprivation | |
| model 3 | Hospital Rural | Ref) |  | 2.30(0.19) ^***^ | 2.29-2.32 |
|  | Clinic Rural | 46.09(6.41) ^***^ | 46.09-46.76 | 1.68(0.12) ^***^ | 1.67-1.69 |
|  | General Hospital Rural | 66.37(7.98) ^***^ | 65.9-66.86 | 0.98(0.03) ^***^ | 0.98-0.99 |
|  | Hospital Urban | 2.67(0.32) ^***^ | 2.65-2.69 | 1.16(0.05) ^***^ | 1.15-1.17 |
|  | Clinic Urban | 30.76(3.60) ^***^ | 30.54-30.99 | 1.12(0.03) ^***^ | 1.11-1.13 |
|  | General Hospital Urban | 57.61(6.63) ^***^ | 57.19-58.03 | 0.77(0.01) ^***^ | 0.77-0.78 |

***: < 0.001

Estimated marginal means (EMMs) for Urbanicity and healthcare facility in model 2 and model 3 were estimated. And optionally, comparisons or contrasts among them. In order to express the interaction between a continuous variable and two categorical variables, Table 3 expresses the interaction of categorical variables(left), and presents the slope of deprivation at the determined categorical variables and deprivation value (0) (right).

Figure 1. District probability of getting TB treatment support. District TB treatment support probability is estimated by the empirical Bayesian method with (1) model 2 that include the 2-way interaction effect of urbanicity and deprivation, and (2) model 3 that include the 3-way cross-level interaction effect of urbanicity, deprivation, and healthcare facility. To consider the effect of the patient size by district, a weighted regression analysis was performed in which the district estimate was weighted by the number of patients by district in the corresponding group. The relationship between the deprivation, urbanicity and TB treatment support (1-(1)) and the relationship between deprivation, urbanicity, healthcare facility and TB treatment support (1-(2)) were presented as the regression coefficient.

Figure 2. Ratio of unsupported TB patients to facilities according to urbanization and district deprivation. The ratio of the number of patients who did not support TB treatment by district and the number of healthcare facility they used was estimated according to urbanicity and deprivation. To consider the effect of the patient size by district, a weighted regression analysis was performed in which the district estimate was weighted by the number of patients by district in the corresponding group. The relationship between the deprivation, urbanicity and ratio of unsupported TB patients to facilities were presented as the regression coefficient.

Supplemental Material

Table S1. Empirical Bayesian estimates using other fixed level values

|  |  | Median | IQR | Range |
| --- | --- | --- | --- | --- |
| 30~64yr, Women, Pulmonary TB(smear+), Korean | Rural | 72.8 | 70.5-74.5 | 64.9-80.6 |
|  | Urban | 80.7 | 74.3-83.0 | 62.9-86.6 |
| 30~64yr, Men, Pulmonary TB(smear+), Korean | Rural | 69.9 | 67.5-71.8 | 61.6-78.3 |
|  | Urban | 78.5 | 71.6-81.0 | 59.6-84.9 |
| ≥ 65, Men, Pulmonary TB(smear+), Korean | Rural | 66.4 | 63.9-68.1 | 55.7-75.4 |
|  | Urban | 75.6 | 68.1-78.4 | 55.7-82.7 |
| 30~64yr, Men, Pulmonary TB(smear+), Migrant | Rural | 63.6 | 61.0-65.7 | 54.7-73.1 |
|  | Urban | 73.3 | 65.4-76.2 | 52.6-80.9 |
