# Supplementary material for: Evaluation of the accessibility and its equity of the national public-private mix program for tuberculosis in Korea: a multilevel analysis
Source: Epidemiol Health. 2022 Dec 7;45:e2023002. doi: 10.4178/epih.e2023002 (PMC10266928; doi:10.4178/epih.e2023002)
Supplement: Supplementary Material 1. — TB-PPM program in South Korea [file epih-45-e2023002-Supplementary-1.docx]

**Supplemental Material 1**

**TB-PPM program in South Korea**

Korean NTP dispatched TB-specialty nurses to 22 general hospitals that have treated more than 250 cases of TB per year since 2009 and operated a PPM demonstration program that provides PPM coverage, including prescription supervision for TB patients.

In 2011, NTP expanded the PPM program across the country to include 252 public health centers (PHCs) and 120 healthcare providers (HCPs) that treat more than 100 cases of TB. PPM program included some clinics affiliated with the Korean National Tuberculosis Association in clinic-level PPM HCPs.

The PHCs not only play the role of regional TB management authorities but also serve as the primary HCPs for TB care. NTP operated this program as a "referral to participating HCPs" centered on general hospitals with large caseloads.

PPM program remained a hospital-based system supporting primarily urban areas, though the number of participating hospitals increased to 22 in 2009, 120 in 2011 due to the nationwide expansion of the program [1], 127 in 2015, and 184 as of 2022 [24].

Of the HCPs that have treated TB patients, 133 out of 365 general hospitals (36.4%), 4 out of 1395 hospitals (0.3%), and only 2 out of 1189 clinics (0.2%) participated in the PPM program,
