# Supplementary material for: Evaluation of the accessibility and its equity of the national public-private mix program for tuberculosis in Korea: a multilevel analysis
Source: Epidemiol Health. 2022 Dec 7;45:e2023002. doi: 10.4178/epih.e2023002 (PMC10266928; doi:10.4178/epih.e2023002)
Supplement: Supplementary Material 2. — Empirical Bayesian estimates using other fixed level values [file epih-45-e2023002-Supplementary-2.docx]

**Supplemental Material 2**

Empirical Bayesian estimates using other fixed level values

|  |  | Median | IQR | Range |
| --- | --- | --- | --- | --- |
| 30~64yr, Women, Pulmonary TB(smear+), Korean | Rural | 72.8 | 70.5-74.5 | 64.9-80.6 |
|  | Urban | 80.7 | 74.3-83.0 | 62.9-86.6 |
| 30~64yr, Men, Pulmonary TB(smear+), Korean | Rural | 69.9 | 67.5-71.8 | 61.6-78.3 |
|  | Urban | 78.5 | 71.6-81.0 | 59.6-84.9 |
| ≥ 65, Men, Pulmonary TB(smear+), Korean | Rural | 66.4 | 63.9-68.1 | 55.7-75.4 |
|  | Urban | 75.6 | 68.1-78.4 | 55.7-82.7 |
| 30~64yr, Men, Pulmonary TB(smear+), Migrant | Rural | 63.6 | 61.0-65.7 | 54.7-73.1 |
|  | Urban | 73.3 | 65.4-76.2 | 52.6-80.9 |
